# Optimization of a parallel permutation testing function for the SPRINT R package

**DOI:** 10.1002/cpe.1787

**Published:** 2011-06-23

**Authors:** Savvas Petrou, Terence M Sloan, Muriel Mewissen, Thorsten Forster, Michal Piotrowski, Bartosz Dobrzelecki, Peter Ghazal, Arthur Trew, Jon Hill

**Affiliations:** 1 Edinburgh Parallel Computing Centre, University of EdinburghEdinburgh, EH9 3JZ, UK; 2 Division of Pathway Medicine, University of Edinburgh Medical SchoolEdinburgh, EH16 4SB, UK; 3 Applied Modeling and Computation Group, Department of Earth Science and Engineering, Imperial CollegeLondon, SW7 2AZ, UK

**Keywords:** HPC, MPI, Permutation, Microarray, R, SPRINT

## Abstract

The statistical language R and its Bioconductor package are favoured by many biostatisticians for processing microarray data. The amount of data produced by some analyses has reached the limits of many common bioinformatics computing infrastructures. High Performance Computing systems offer a solution to this issue. The Simple Parallel R Interface (SPRINT) is a package that provides biostatisticians with easy access to High Performance Computing systems and allows the addition of parallelized functions to R. Previous work has established that the SPRINT implementation of an R permutation testing function has close to optimal scaling on up to 512 processors on a supercomputer. Access to supercomputers, however, is not always possible, and so the work presented here compares the performance of the SPRINT implementation on a supercomputer with benchmarks on a range of platforms including cloud resources and a common desktop machine with multiprocessing capabilities. Copyright © 2011 John Wiley & Sons, Ltd.

## 1. INTRODUCTION

Analyses of post-genomic data are requiring increasingly large amounts of computational processing power and memory to complete. A popular free statistical software package for carrying out this data analysis is R [1]. At the University of Edinburgh, Edinburgh Parallel Computing Centre along with the Division of Pathway Medicine, designed and built a prototype package for R, called Simple Parallel R Interface (SPRINT) [2, 3], which parallelized a key statistical correlation function of important generic use to machine learning algorithms (clustering, classification) in genomic data analysis. This prototype successfully demonstrated that parallelized statistical functions could be interfaced with R, providing biologists with an easy route into High Performance Computing (HPC).

The aim of SPRINT is to require minimal HPC knowledge, minimal changes to existing R scripts, and yet give maximum performance. The package provides an *interface* to HPC and a *library* of parallel functions. This paper focuses on the work undertaken to extend the library through the addition of a parallel permutation testing function.

The SPRINT project carried out a user requirements survey [4] to collect information from the bioinformatics community on which R functions are causing bottlenecks when processing genomic data as well as those that are currently intractable on standard desktop machines. Some of these functions are suited to large-scale distributed-memory machines, and this paper is focused on implementing one of these functions. In the SPRINT user requirements survey, respondents were asked to list the five R functions they would consider most useful for inclusion in a parallel R function library. Permutation functions were the 2nd most requested class of functions with the *mt.maxT* function [5] from the multtest package [6] being one of the most widely used of these.

This paper elaborates on the work presented in Petrou *et al.* [7] by presenting further benchmarking results for a parallel implementation of *mt.maxT* on a range of compute platforms from multi-core PCs through to the cloud. In [7], Petrou *et al.* undertook a limited performance analysis that established that *pmaxT*, the SPRINT parallel implementation of the permutation testing function *mt.maxT*, scales on a supercomputer. However, not all life scientists have access to such an expensive and specialist platform for their research. The work presented here therefore seeks to establish if SPRINT's *pmaxT* can produce performance benefits across a range of platforms, some of which are more likely to be accessible to life scientists and have different hardware architectures to the supercomputer in [7]. In particular, a cloud environment is included because, subject to security concerns, it potentially offers life scientists the opportunity of on-demand access to supercomputing level capabilities without expensive acquisition, ongoing maintenance and power requirements. There are, however, potential networking issues in clouds that could have a detrimental impact on performance of SPRINT's *pmaxT*, and so this paper comments further on these in the context of the collected results.

To allow a valid comparison, the results from [7] are therefore presented alongside the results from the additional benchmarking platforms undertaken here.

The following section in this paper describes how SPRINT and its architecture relate to similar R packages for exploiting parallelism. Subsequent sections describe the existing serial implementation of permutation testing, *mt.maxT*, and its SPRINT parallel counterpart *pmaxT*. Following this, the benchmarking of *pmaxT* on a variety of systems is described, and the results discussed. Finally, the paper offers some conclusions on the applicability of *pmaxT* and possible future work.

## 2. SPRINT AND RELATED WORK

The R package, as released currently, has no built-in parallel features. A few open source groups from the R community have developed various packages [8, 18, 19, 20, 21, 22] in an effort to enable R to run in parallel. These packages can execute simple parallel functions with no data dependencies. The primary objective of SPRINT is to contribute to this effort by adding functionality beyond the limits of the available parallel packages.

The existing packages offer tools for applying various forms of parallelism in R codes. However, many R functions, like *mt.maxT*, are implemented in the C language with only their interface implemented in R. In these circumstances, the most commonly used approach to exploit parallelism is to perform multiple executions on subsets of the dataset, the iteration count or both. By doing so, the memory demand and the run time of each individual execution are reduced to a reasonable level. Although this workaround can be applied in many cases, the partial data produced still have to be reduced and processed in order to be transformed into the expected output.

Hill *et al.* [2], in describing SPRINT also explained how SPRINT differs from the other parallel implementations of R that are available. In SPRINT, R functions are implemented at the C level (either directly in C or called via a C harness) and executed in parallel. Using SPRINT, there are minimal changes to the R code that a user writes. The results are reduced and transformed to the expected output before they are returned to the user, thus requiring no further processing by the user. In addition, processes can communicate and exchange data, which enable data to have dependencies.

Figure 1 from Dobrzelecki *et al.* [9], shows the latest architecture of SPRINT. Since its original description in Hill *et al.* [2], the SPRINT architecture has been extended further to allow the underlying workers to exploit also the existing serial R functionality as well as functionality written specifically in C and Message Passing Interface (MPI) [10].

**Figure 1:**
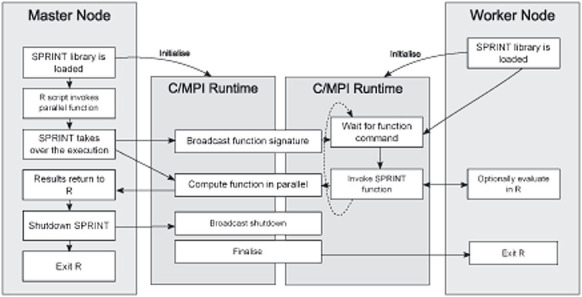
The SPRINT framework architecture as described in [9].

As described in Dobrzelecki *et al.* [9], all participating processes instantiate the R runtime, load the SPRINT library and initialise MPI with the workers entering a waiting loop until receipt of an appropriate message from the master. The master evaluates the users’ R script; if this includes a parallel function from the SPRINT library, the workers are notified, the data and computation distributed and the workers collectively evaluate the function, returning their results to the master [9]. The master collects the results from the workers, performs any necessary reduction and returns the result to R [9].

Allowing SPRINT workers to also exploit existing serial R functionality means that when appropriate, the data, iteration count or both can be partitioned by SPRINT across the workers, processed by the serial R functionality with the results collected and reduced by the master, and the final result returned to R. From a user perspective, this means that there is no need to perform the additional steps associated with manual partitioning of data or iterations and the subsequent manual collection and reduction of results. Mitchell *et al.* [11] describe the implementation of the R Random Forest Classifier in SPRINT using this approach.

To achieve the best performance, the SPRINT implementation of permutation testing described in this paper is implemented directly in C and MPI on the workers rather than these workers using serial R.

## 3. THE PERMUTATION FUNCTION

### 3.1. The serial version: mt.maxT

In statistical analysis, a permutation test is one of the methods used for computing the statistical significance of data. It is a non-parametric statistical method that avoids using assumptions about the underlying data by recomputing a given test statistic on large numbers of randomly or completely permuted versions of the input data. By comparing the results from this *null distribution* of randomized data to a single result obtained on the actually observed data, statistical significance (*p*-value) can be determined. This procedure is carried out for each row (e.g. gene) in a data matrix and results in n *p*-values if there are n rows. The R *mt.maxT* function computes the adjusted *p*-values for step-down multiple testing procedures [5], as they are described in Westfall and Young [12].

The function supports two types of permutation generators. A *random* permutations generator (Monte-Carlo sampling) and a *complete* permutations generator. Moreover, it supports six different methods for statistics, used for testing the null hypothesis of no-association between the class labels and the variables. For all the methods, both generators are implemented. Furthermore, for all combinations of method/generator, the user can either choose to save the permutations in memory before the computations take place or compute them on the fly. Considering all these options, there are 24 possible combinations of generator/method/store.

The six supported statistics methods are the following:

- **t**: Tests based on a two-sample Welch *t*-statistics (unequal variances).
- **t.equalvar**: Tests based on two-sample *t*-statistics with equal variance for the two samples.
- **Wilcoxon**: Tests based on standardized rank sum Wilcoxon statistics.
- **f**: Tests based on *f*-statistics.
- **Pair-t**: Tests based on paired *t*-statistics.
- **Block-f**: Tests based on *f*-statistics, which adjust for block differences.

Four of the statistics methods (t, t.equalvar, Wilcoxon and f) are similar in nature and use the same implementation of generators/store. In addition, for *complete* permutations, the function never stores the permutations in memory. Although the option is available, it is implemented using the on-the-fly generator of permutations. For the *Block-f* statistics method, the permutations are never stored in memory because of the huge amount of permutations produced. The option is available, but the code is again implemented using the on-the-fly generator. The distinct combinations of generator/method/store the function implements are therefore eight.

### 3.2. The parallel version: pmaxT

Parallelism can be introduced in two ways. The first approach is to divide the input dataset and perform all permutations on the partial data. The second approach is to distribute the permutations so that each process has the entire dataset but executes only a few of the requested permutations.

Because the calculation of the adjusted *p*-values in the Westfall–Young method requires data from all the rows in the input dataset, the division of input data approach to parallelism necessitates a synchronization of all processes at each permutation. Whereas this first approach does offer the possibility of processing larger datasets, this synchronization will have a significant impact on the performance of a parallel implementation. For bioinformaticians using the R *mt.maxT* function, the input dataset will generally fit in the available processor memory. Instead, it is the elapsed time of the number of permutations they wish to execute that is the limiting factor to their analyses. Essentially, these users wish to execute more permutations to better validate their experimental results, but the time cost of doing sufficient permutations is prohibitive.

Our implementation has therefore taken the second approach and so instead divides the permutation count into equal chunks and assigns them to the available processes, each of which has access to the entire dataset. At the end of the computational kernel, each process will have gathered a part of the observations needed to compute the raw and adjusted *p*-values. These observations are gathered on the master process where the *p*-values are computed and returned to R.

**Figure 2:**
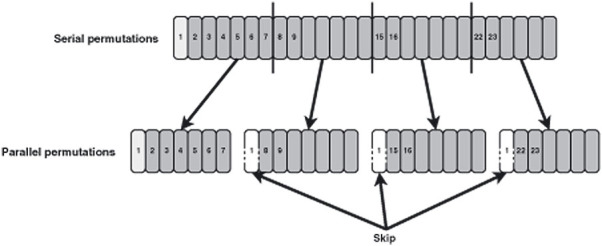
How permutations are distributed among the available processes.

To be able to reproduce the same results as the serial version, the permutations performed by each process need to be selected with caution. Figure 2 shows how the permutations are distributed among the available processes. The first permutation depends on the initial labelling of the columns, and it is thus *special*. This permutation only needs to be taken into account once by the master process. The remaining processes skip the first permutation (from both the complete and random generators). Moreover, the generators need to be forwarded to the appropriate permutation. The interface of the generators was altered to accommodate an additional variable to the initialization function. Depending on the value of this variable, the generators *skip* a number of cycles and forward to the appropriate permutation.

The parallel implementation executes the following steps:

- **Step 1:** The master process executes a pre-processing step to check the input parameters and transform a few to the format expected by the C code.
- **Step 2:** All processes, apart from the master process, allocate memory to accept the input parameters. The master process broadcasts the lengths of the string parameters first. All the scalar integer options are also broadcast for convenience. These values are received in a statically allocated buffer vector. All dynamically allocated memory needed is then allocated, initialized and checked.
- **Step 3:** A global sum is performed to synchronize all processes and ensure that the necessary memory is allocated. This memory includes the input parameters, the input data and the memory to store the final results.
- **Step 4:** Each process computes how many permutations it needs to calculate and initializes its generator to the appropriate permutation. Then it computes the local observations.
- **Step 5:** The master process gathers the partial observations and computes the raw and adjusted *p*-values. The computed values are saved in a memory space allocated by the pre-processing script at the R level. This is necessary in order for the values to be returned to R when the computations are finished.
- **Step 6:** All processes free their dynamically allocated memory.

The interface of the *pmaxT* is identical to the interface of *mt.maxT*. All functionality was sucessfully ported to the parallel version:


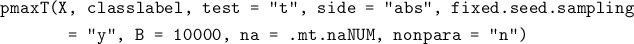


Compared with:


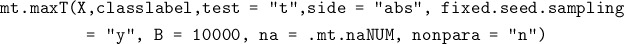


Parameters test, side, fixed.seed.sampling, B, na and nonpara are optional. If omitted, the default values shown above are used. The description of the input parameters follows:

- **X** : The input dataset array.
- **classlabel** : The class labels of the columns of the input dataset.
- **test** : The method for statistics, used for testing the null hypothesis.
- **side** : The type of rejection region. Available options are abs for absolute difference, upper for the maximum and lower for the minimum.
- **fixed.seed.sampling** : The choice between computing the permutations on the fly or save all permutations in memory prior to computations. Available options are y (yes) for the on-the-fly generator and n (no) for storing them in memory.
- **B** : The number of permutations. When this value is set to 0, the code will try to perform the complete permutations of the data. In case the complete permutations exceed the maximum allowed limit, the user is asked to explicitly request a smaller number of permutations.
- **na** : The code for missing values. All missing values will be excluded from the computations.
- **nonpara** : The option for non-parametric test statistics. Available options are y for yes and n for no.

## 4. BENCHMARKS

### 4.1. High Performance Computing systems

The SPRINT team have previously reported [7] benchmarking this parallel function on just the UK National Computing Service HECToR [13]. In this paper, this benchmarking is extended to different systems in order to observe how it scales on a variety of HPC resources from supercomputers down to quadcore PCs. Given the popularity of accelerator technologies, the obvious question to ask is why are platforms with GPUs or similar accelerators not included in the benchmark systems. The answer to this betrays the current portability and standards issues associated with such accelerators - parallelism in SPRINT is based on the recent incarnations of the MPI standard [10]. This obviously means that SPRINT can run without modification on a variety of cluster configurations and shared memory multi-processor platforms. Moreover, using MPI allows SPRINT to tackle more than the embarrassingly parallel problems that accelerators such as GPUs are optimized for [14].

The choice of benchmark systems allows testing of the hypothesis that the SPRINT architecture enables a life scientist to scale up their existing R permutation analyses from their local PC to shared memory multi-core platforms, clouds and supercomputers.

The hardware specifications of the systems used at the time the benchmarks were executed follow:

*HECToR* The UK National Supercomputing service [13], Cray XT system HECToR, comprised 1416 compute blades, each with four quad core processor sockets. The CPUs were AMD 2.3 GHz Opteron chips with 8 GB of memory. This gave a total of 22 656 active cores with a total of 45.3 TB of memory and a theoretical peak performance of 208 TFLOPS.

*ECDF* The Edinburgh Compute and Data Facility [15], known as Eddie, was a Linux compute cluster. It comprised 128 IBM iDataPlex servers, each server node had two Intel Westmere quad-core processors sharing 16 GB of memory.

*Amazon EC2* The Amazon Elastic Cloud (EC2) [16] resource is a virtual machine computing environment. The user requests a number of virtual machines to be spawned, called instances, and can use them to set up and run applications in parallel. A variety of system configurations for these virtual machines are available. The machine used for the permutation function benchmarks had the following hardware specifications:

- 15 GB memory
- 8 EC2 Compute Units (4 virtual cores with 2 EC2 Compute Units each)
- 1 690 GB instance storage (4 x 420 GB plus 10 GB root partition, high I/O performance)
- 64-bit platform

*Ness* Edinburgh Parallel Computing Centre's internal symmetric multi-processing system, Ness, consisted of 16 dual-core 2.6 GHz AMD Opteron (AMD64e) processors divided into two boxes of 16 cores each. Each core had 2 GB of memory associated with it, giving a total of 32 GB per box, shared between 16 cores.

*Quad-core desktop* The last system was a quad-core Intel Core2 Quad Q9300 personal desktop machine with 3 GB of memory. Biostatisticians often execute small analyses on such systems. Benchmarking a desktop multicore machine showed if the function can be efficiently used in a mini-HPC system with multiprocessing capabilities.

### 4.2. Using R and SPRINT

In order to use SPRINT, a user needs the latest version of R and an MPI library (that supports MPI-2) already installed on the system. SPRINT is distributed as a single compressed tar file, which contains all the source files needed for installation. The installation is then carried out with a standard R command

R CMD INSTALL

from the directory containing SPRINT. This scans the system for all known MPI compilers, checks if the identified MPI implementation has MPI-2 support, compiles and runs a small MPI program to test all the settings. After a successful installation, a user can utilize the processing power of multiple CPUs by executing an R script containing SPRINT functions via mpiexec or mpirun (depending on MPI implementation). For example,

mpiexec n NSLOTS R no-save f SPRINT_SCRIPT_NAME

### 4.3. Benchmarks results for pmaxT

The benchmarks were executed for process counts of 1 up to 512, where supported. Of the benchmark systems, the authors only had sole access to the quad-core desktop. This meant that if other users were utilizing a disk heavily that was also being used to output SPRINT benchmark results then the average run-time for the SPRINT benchmarks is significantly skewed by such behaviour. For this reason, the values shown in the tables are the minimum measured timings obtained from five independent executions.

**Figure 3:**
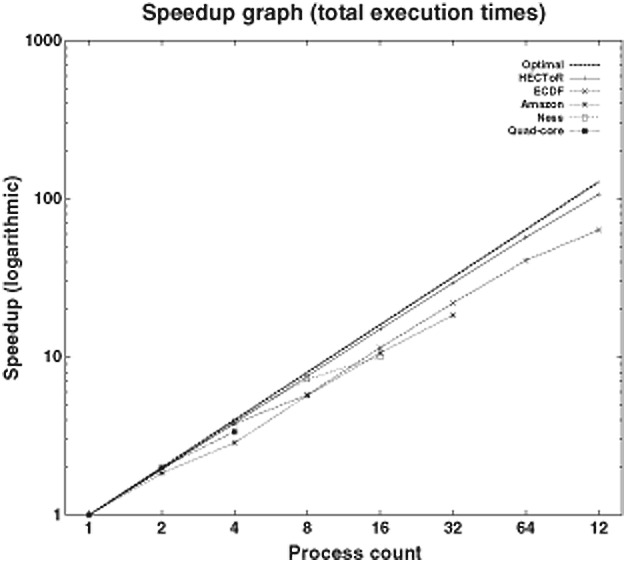
*pmaxT* speed-up on the various systems.

The benchmark results for Tables I–V are for the execution of a permutation count of 150 000 on a dataset of 6 102 rows (genes) and 76 columns (samples), a reasonably sized gene expression microarray after pre-processing to remove non-expressed genes. These tables show the time spent on each of the five main sections of the function. Figure 3 shows how the parallel version scales on the different systems and how it compares to the optimal speedup.

**Table I:** Profile of *pmaxT* implementation (HECToR)

Process count	Pre processing (s)	Broadcast parameters (s)	Create data (s)	Main kernel (s)	Compute *p*-values (s)	Speedup	Speedup (kernel)
1	0.260	0.001	0.010	795.600	0.002	1.00	1.00
2	0.261	0.004	0.012	406.204	0.884	1.95	1.95
4	0.259	0.009	0.013	207.776	0.005	3.82	3.82
8	0.260	0.013	0.013	104.169	0.489	7.58	7.63
16	0.259	0.015	0.013	51.931	0.713	15.03	15.32
32	0.259	0.017	0.013	25.993	0.784	29.40	30.60
64	0.259	0.020	0.013	13.028	0.611	57.11	61.06
128	0.259	0.023	0.013	6.516	0.662	106.48	122.09
256	0.260	0.024	0.013	3.257	0.611	190.99	244.27
512	0.260	0.028	0.013	1.633	0.606	313.09	487.20

**Table II:** Profile of *pmaxT* implementation (ECDF)

Process count	Pre processing (s)	Broadcast parameters (s)	Create data (s)	Main kernel (s)	Compute *p*-values (s)	Speedup	Speedup (kernel)
1	0.157	0.000	0.003	467.273	0.000	1.00	1.00
2	0.163	0.002	0.003	234.848	0.000	1.99	1.99
4	0.162	0.003	0.004	123.174	0.000	3.79	3.79
8	0.159	0.004	0.005	79.576	1.217	5.77	5.87
16	0.158	0.032	0.005	39.467	1.224	11.43	11.84
32	0.164	0.072	0.005	19.862	1.235	21.91	23.53
64	0.157	0.072	0.005	9.935	1.297	40.77	47.03
128	0.162	0.086	0.007	5.813	1.304	63.40	80.38

**Table III:** Profile of *pmaxT* implementation (Amazon EC2)

Process count	Pre processing (s)	Broadcast parameters (s)	Create data (s)	Main kernel (s)	Compute *p*-values (s)	Speedup	Speedup (kernel)
1	0.272	0.000	0.006	539.074	0.000	1.00	1.00
2	0.271	0.004	0.009	291.514	0.005	1.84	1.84
4	0.273	0.011	0.014	187.342	0.043	2.87	2.87
8	0.278	0.880	0.014	90.806	2.574	5.70	5.93
16	0.268	1.735	0.022	43.756	4.983	10.62	12.32
32	0.270	2.917	0.019	22.308	3.834	18.37	24.16

**Table IV:** Profile of *pmaxT* implementation (Ness)

Process count	Pre processing (s)	Broadcast parameters (s)	Create data (s)	Main kernel (s)	Compute *p*-values (s)	Speedup	Speedup (kernel)
1	0.393	0.000	0.010	852.223	0.000	1.00	1.00
2	0.467	0.007	0.012	443.050	0.001	1.92	1.92
4	0.398	0.029	0.012	216.595	0.001	3.93	3.93
8	0.394	0.032	0.014	117.317	0.001	7.24	7.26
16	0.436	0.109	0.019	84.442	0.001	10.03	10.09

**Table V:** Profile of *pmaxT* implementation (Quad Core desktop)

Process count	Pre processing (s)	Broadcast parameters (s)	Create data (s)	Main kernel (s)	Compute *p*-values (s)	Speedup	Speedup (kernel)
1	0.140	0.000	0.007	566.638	0.001	1.00	1.00
2	0.136	0.003	0.008	282.623	0.085	2.00	2.00
4	0.135	0.010	0.013	167.439	0.705	3.37	3.38

In addition, benchmarks were executed to measure the amount of time the parallel implementation needs to execute a very high permutation count. Table VI shows the elapsed run times for two differently sized input datasets with increasing permutations counts. All executions for this Table were performed on the HECToR, the UK National Supercomputing Service, with 256 processes. The run times for the serial implementation in Table VI are not actual measured timings but an approximation of the real run time of the original serial R implementation running on a single core. Executions of this with lower permutation counts (1000, 2000 and 3000 permutations) showed a linear increase in run time as the permutation count increases. According to these results the approximated run times were calculated.

**Table VI:** Comparing the elapsed run times of *pmaxT* and the original serial R implementation for processing two datasets of different size with increasing permutation count. The *pmaxT* runs were executed on 256 cores of HECToR whereas the serial R run times are estimates based on smaller permutation counts on a single core

Input array dimension and size (genes ×samples)	Permutation count	Total run time (s)	Serial run time (approximation) (s)
36 612 × 76 21.22 MB	500 000	73.18	20 750 (6 h)
	1 000 000	146.64	41 500 (12 h)
	2 000 000	290.22	83 000 (23 h)
73 224 × 76 42.45 MB	500 000	148.46	35 000 (10 h)
	1 000 000	294.61	70 000 (20 h)
	2 000 000	591.48	140 000 (39 h)

### 4.4. Discussion of benchmark results

As indicated in Tables I–V, the results from the benchmarks show very good scaling behaviour on all systems, not just on the previously reported HECToR [7]. As the number of processes used on all the benchmark systems increases, the amount of time needed by the function reduces linearly. However, for very large process counts, the overhead from broadcasting and transforming the input dataset may consume a significant percentage of the total run time and affect the overall scaling (see the last two columns in Tables I and II).

Looking at the scaling of the computational kernel alone in Tables I–V, ignoring any communications, it can be seen how well the implementation performs as the process count increases again on all the benchmark systems. When the work load of the computations is sufficiently high, the amount of time spent exchanging and transforming data will be small enough that it does not affect the overall scaling. This is particularly apparent when comparing the total speed-up and kernel speed-up on HECToR (Table I), the ECDF (Table II) and the Amazon cloud environment (Table III) where at lower processor counts, the total and kernel speed-ups are almost matching but start to diverge more and more at higher process counts.

It is also noticeable that a drop-off in speed-up occurs on both ECDF and the Amazon Cloud at process counts of 4–8 and 2–4, respectively. This is likely to correspond to the memory bus bandwidth because a node on the ECDF consists of two quadcores sharing memory.

When considering the size of the input dataset, Table VI shows how doubling the input dataset size results in a close to doubling of the elapsed time for various permutation counts on HECToR atleast.

Conclusions about the performance and importance of the network can be drawn by examining two of the timed sections, the *Broadcast parameters* and the *Compute p-values*. These two sections perform collective communications between all processes, and their performance relies heavily on the underlying network. HECToR, the UK National Supercomputing Service, uses a high speed proprietary Cray interconnect network, the SeaStar2. By examining the times measured, it can be seen that the additional time needed by the two sections when the process count doubles, increases linearly. Similar behaviour is observed for the ECDF system. This resource's interconnect uses a Gigabit Ethernet network. Both interconnects have high bandwidths and low latencies, which ensure that communications are performed efficiently.

Regarding the cloud environment, the Amazon EC2 instances are connected using a virtual ethernet network with no guarantees on bandwidth or latency and therefore performs poorly when the process count increases. When new instances are added to the pool, and more communications are needed, the additional time increases dramatically. The greater divergence between total and kernel speed-up at a lower process count is further evidence of this.

In contrast, the quad-core desktop and Ness are shared memory systems and use the main memory as their interconnect. The communication performance of these two machines is, in general, very good because of the high bandwidth and low latency of the memory. However, other applications executing on the same system can affect this performance depending on their memory usage.

Another observation is the noticeable difference in the computational kernel times between resources when only one process is used. The architecture of the CPU plays a major role on how fast the computations are performed. Newer architectures can perform better than older ones. For example, ECDF is much faster than HECToR for a single process execution. The difference is reduced when more processes are used, most likely because of the reduced work load per process. Newer architectures can handle higher work loads more efficiently and can perform better using lower process counts.

As explained in [7], the main challenge for the permutation testing function is the permutation count as shown in Table VI. As the count of requested permutations increases, the run time becomes excessively costly. The parallel implementation distributes the permutations among all available processes, allowing a high permutation count to be performed in a reasonable run time. When the permutations are generated on the fly, the implementation demands no extra memory in order to perform a higher permutation count. As long as the input dataset can be stored in memory, the implementation can execute a permutation count only limited by the precision of the underlying CPU architecture.

Furthermore, the faster execution times of the parallel implementation helps reduce the risk of failures. Long executions are at higher risk of system failures, thus an implementation that performs the same amount of work faster is preferred.

The benchmark results clearly show that for permutation testing using R and the SPRINT package, a life scientist can benefit from high performance computing on a variety of compute platforms without requiring specialized parallel programming skills. Even in cloud environments where bandwidth and latency are not guaranteed, the inherent scalability of the algorithm still means that reasonable performance gains can be had.

## 5. CONCLUSIONS

This paper presents the benchmark results collected from a range of platforms for the SPRINT parallel implementation of an R permutation testing function. This implementation shows close to optimal scaling on the UK National Supercomputing Service. Results presented from other platforms show sufficient speed-up that reasonable performance gains can be had even in environments where network latency can impact.

The SPRINT implementation is able to perform a much higher permutation count within a reasonable execution time. It therefore offers a generic method that can be applied to newer generation microarrays, like Affymetrix Exon Arrays [17]. These arrays have a minimum feature count of around 280 000 and a maximum of around 5 million that need to be tested statistically.

The speed-up in results across the benchmark systems offers a route for life scientists to scale up their analyses based on the infrastructure available to them without having to change their workflows. Using the same permutation testing implementation, life scientists can exercise and refine their workflows on lower end, less expensive platforms before executing more ambitious and potentially costly runs on high-end facilities.

## 6. FUTURE WORK

Given the feedback provided in the SPRINT user requirements survey [4], the obvious areas for future work for the SPRINT package generally are in the addition of more parallelized functions. More specifically considering the parallel implementation of the permutation testing function while this performs well, a few additional changes could be made to further improve it. For example:

1. Better support for fault tolerance and checkpointing; whereas this is not available in the existing serial R implementation, this may be of increasing importance as life scientists wish to perform even more tests on ever larger datasets. As mentioned previously, the parallel implementation does go some way to addressing this by enabling analyses to be repeated more quickly just by being faster, but it is likely that more specific support will be required in the future.
2. The current implementation performs an array transposition on the input dataset. For this transformation, a new array is allocated. Algorithms for in-place non-square array transposition exist that are able to perform this step without the need for additional memory.
3. The *string* input parameters can be replaced with scalar integer values before they are broadcast to all processes. Scalar parameters are easier and faster to broadcast and handle.
